# Pelvic and spinal postural changes between standing-sitting positions following lumbosacral fusion: a pilot study

**DOI:** 10.1007/s00264-022-05365-6

**Published:** 2022-03-09

**Authors:** Thomas Borgeaud, Jean-Charles Le Huec, Antonio Faundez

**Affiliations:** 1 Faculty of Medicine, Université de Genève (UNIGE), Rue Michel-Servet 1, 1206 Geneva, Switzerland; 2 Polyclinique Bordeaux Nord, Centre Vertebra, 15 rue Boucher, 33000 Bordeaux, France; 3 Hôpital de La Tour, Av. J.-D.-Maillard 3, 1217 Meyrin, Switzerland; 4 Department of Surgery, Geneva University Hospitals (HUG), Rue Gabrielle-Perret-Gentil 4, 1205 Geneva, Switzerland

**Keywords:** Adult spinal deformity (ASD), Proximal junction kyphosis (PJK), Proximal junction failure (PJF), Sagittal balance, Sitting position

## Abstract

**Purpose:**

Prospective pre-operative and post-operative comparative analysis of radiographic spino-pelvic parameters between sitting versus standing positions of patients with LS fusion, to detect adaptation mechanisms around fused spinal segments.

**Methods:**

Sixteen patients aged 53.9 ± 15.9 who underwent LS fusion between L3 and S1 were extracted from the database of an ongoing prospective study. Different spino-pelvic parameters were evaluated on full spine X-rays, standing, then sitting straight. Parameters were compared pre-operative versus post-operative, and on standing versus sitting X-rays.

**Results:**

Preliminary results revealed a significantly greater pre-operative pelvic tilt (PT) in sitting than standing posture, (*p* = 0.020) but not in post-operative (*p* = 0.087). After surgery, PT was lower in sitting compared to pre-operative (*p* = 0.034) but not in standing (*p* = 0.245). L4–S1 lordosis was lower in sitting than standing in pre-operative (*p* = 0.014) and post-operative (*p* = 0.021). Surgery decreased segmental lordosis above the fusion (PSL, proximal sagittal lordosis) in sitting (*p* = 0.039) but not in standing (*p* = 0.193). No significant differences in thoracic kyphosis (TK) were observed. Fusions down to L5 versus S1 showed no significant differences for PT and PSL, neither in sitting versus standing, nor pre-operative versus post-operative.

**Conclusion:**

Before fusion, compared to standing, PT increases in sitting straight posture (pelvic retroversion), and the lumbar spine adapts by decreasing its lordosis, mainly at L4–S1. After fusion, the segments adjacent to the instrumented section, adapt in flexion at lumbosacral and thoracolumbar junctions, i.e. just below and above (PSL). This might have mechanical implications for the occurrence of adjacent segment disease.

## Introduction

The vast majority of clinical studies dealing with adult spinal deformities (ASD) and degenerative pathology are based on static radiographs of the spine, in standing posture. Patients surgically treated for ASD do obviously not spend their whole daily life in standing posture, and many of them are probably more often adopting the sitting posture after a certain age (we have not found recent reports on the prevalence of sitting position in the elderly population). In any case, there is a repetitive posture change, a dynamic aspect, that current studies have yet not really appraised, and might be a cause of junctional mechanical complication.

In normal subjects, several publications have shown that, when changing from standing to sitting position, the pelvis retroverts and the lumbar spine adapts by decreasing its lordosis [1–4]. The decrease in lordosis varies according to the type of sitting posture: Hey et al. found in a study of normal subjects comparing standing straight versus sitting straight postures, that the PT increases by at least 50% (retroversion) and consequently the lumbar lordosis decreases by an average of almost 25° [4].

As of today, there is no study, to our knowledge, that has analysed postural adaptations between standing and sitting, of patients undergoing lumbosacral fusion.

Our hypothesis is that patients with lumbosacral fusion need to compensate the loss of mobility in fused segments, by flexion above and below the fused segments. This might have an influence on the occurrence of junctional deterioration, depending on the segmental angulation of adjacent levels: junctional kyphosis (acute appearance, within six to eight weeks post-operative) or adjacent segment disease (chronic process > 3 months).

We also hypothesise that patients with fusion down to L5 have different compensation/adaptative abilities than patients fused down to S1 (last mobile segment): if L5–S1 is not fused, patients can compensate by flexing forward at this segment and decrease the mechanical stresses on the more proximal levels.

## Material and methods

A series of 16 patients have been extracted from a prospective electronic database and patients gave their approval for storage of their clinical and radiological data according to the Declaration of Helsinki and approved by ethics committee of the Geneva University Hospitals (reference CCER 11–113).

**Fig. 1:**
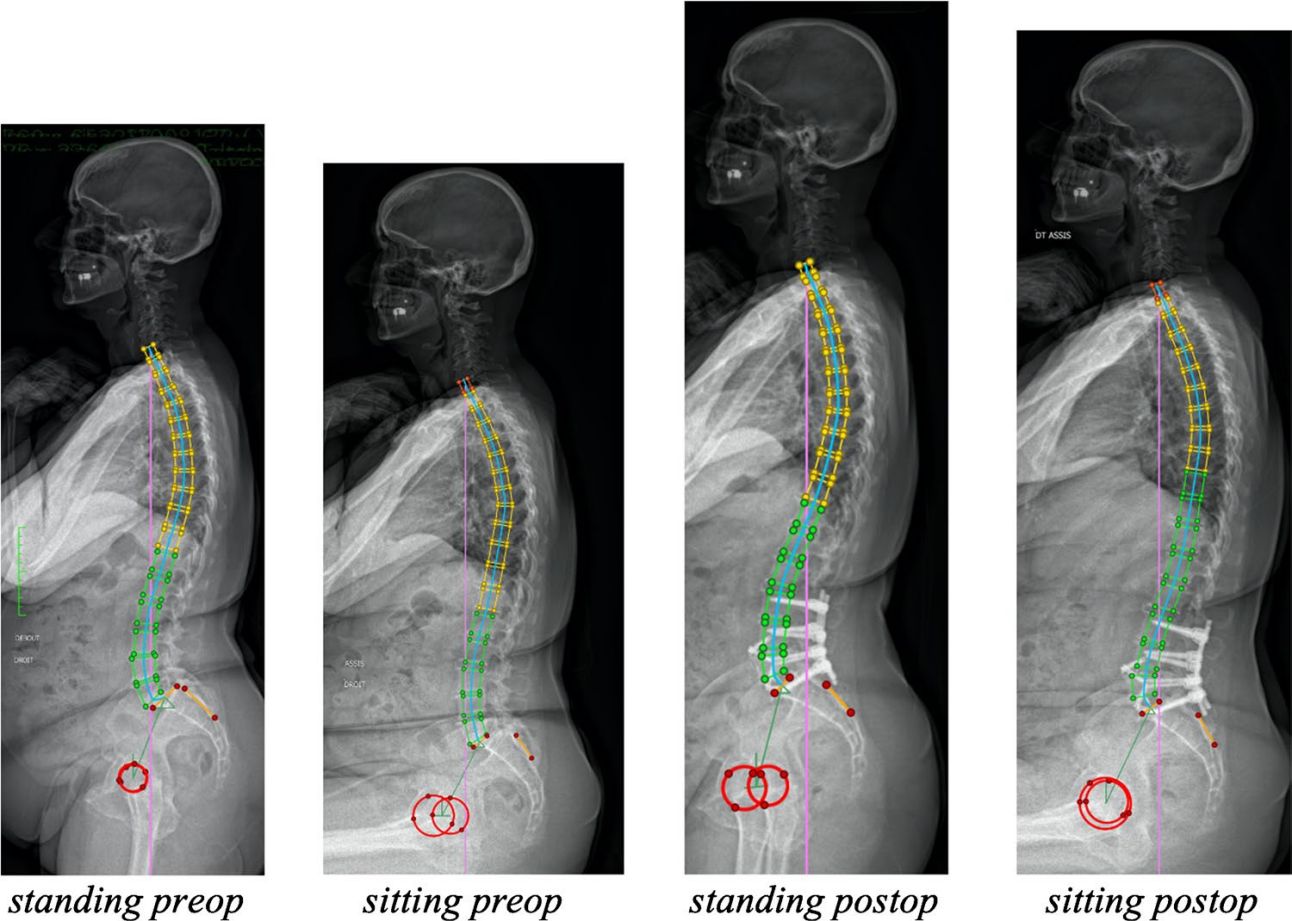
Radiographs by EOS and reconstruction with Keops® software

In this prospective study, we analysed pre-operative and post-operative (6 weeks post-operative and latest follow-ups) radiographs (from 2017 to 2020) in standing versus sitting straight postures for each patient, using the EOS system and a web-based sagittal balance analysis software (Fig. 1). The goal of the study was to evaluate postural changes between standing and sitting position for these patients and no clinical data has been considered at this point, although it is being collected and will be subject to analysis at a later stage.

Standard spino-pelvic parameters were measured and compared between pre-operative and post-operative, as recommended in the literature [5].

We measured pelvic incidence (PI), pelvic tilt (PT), sacral slope (SS), C7 sacral vertical axis (C7 SVA), lumbar lordosis (GLL), L4–S1 lordosis (LLL), L5–S1 lordosis, lumbar apex position, segmental lordosis immediately above the fusion (proximal segmental lordosis, PSL) and below (if applicable) the fusion construct, and thoracic kyphosis T1–T12 (TK). For PSL, we classified it in three different morphologies: lordotic, neutral or kyphotic. The Cobb angles at the PSL were automatically measured by the web-based sagittal balance software, after we manually delimited the vertebral endplates. All the measurements were done by the first author, supervised and validated by the last author.

Patients with fusion to S1 have been analysed separately from patients with fusion to L5 for PT and PSL post-operatively.

For statistical analysis, we decided to compare medians instead of means for each sagittal parameter due to the small number of patients included in this pilot study. Shapiro–Wilk test was used to determine normality of the distribution for each parameter. For normal distributions, we compared medians using unpaired Student’s *T*-test to analyse standing versus sitting straight position and Wilcoxon-rank-sum test for non-normal distributions. To analyse pre-operative versus post-operative measures, we used respectively paired Student’s *T*-test for normal distributions and Wilcoxon signed-rank test for non-normal ones. All calculations were made with the R® software. *P*-value < 0.05 was considered as statistically significant.

## Results

There were 16 patients included in our pilot study: nine men (56.3%) and seven women (43.8%), mean age of 53 years (range 27–82). Mean follow-up period was 15.9 months (± 9.5). Ten patients had L4–L5 fusion (62.5%) and the six others had fusion down to S1 (37.5%). The most proximal instrumented segment was L3.

No statistically significant differences in PI were observed in pre-operative versus post-operative, neither standing nor sitting straight (*p* > 0.05) (Table 1).

**Table 1:** Pre- and post-operative radiographic measurements stratified by subjects posture

	Standing posture (*n* = 16 patients)				Sitting posture (*n* = 16 patients)				*p-*value
	Mean ± SD	Median	(IQR)	(Range)	Mean ± SD	Median	(IQR)	(Range)	
Pelvic incidence (°)									
Pre-operative	53.8 ± 13.2	50.5	(42.6–64.4)	(38.5–76.3)	55.6 ± 13.0	51.7	(44.4–65.7)	(41.8–80.0)	0.500
Post-operative	53.5 ± 12.4	48.5	(43.7–61.4)	(39.8–77.7)	54.1 ± 13.4	49.7	(45.9–62.5)	(33.1–83.0)	0.780
Improvement	− 0.4 ± 3.1	− 0.1	(− 2.3 to 1.6)	(− 6.6 to 4.4)	− 1.5 ± 4.3	− 0.8	(− 3,7 to 1.8)	(− 10.7 to 6.1)	0.590
*p*-value*	0.744				0.274				
Pelvic tilt (°)									
Pre-operative	18.4 ± 8.2	15.8	(13.0–25.8)	(1.4–30.2)	27.1 ± 10.7	26.7	(19.7–32.2)	(13.7–52.3)	0.020
Post-operative	17.1 ± 6.5	15.6	(12.4–23.6)	(5.7–27.0)	23.3 ± 10.6	24.6	(16.0–30.6)	(6.3–43.8)	0.087
Improvement	− 1,3 ± 4.3	− 0.4	(− 4.4 to 0.8)	(− 8.2 to 9.6)	− 3.8 ± 6.3	− 4.3	(− 8.1 to 0.1)	(− 15.1 to 6.8)	0.163
*p*-value*	0.245	15.8			0.034				
Sacral slope (°)									
Pre-operative	35.5 ± 7.6	34.9	(28.1–41.8)	(25.0–48.3)	28.5 ± 10.6	30.1	(25.2–35.7)	(2.5 to 42.6)	0.062
Post-operative	36.4 ± 8.5	33.0	(30.1–42.2)	(25.6–54.1)	30.8 ± 9.2	29.5	(23.8–37.0)	(16.7 to 48.5)	0.102
Improvement	0.9 ± 3.6	0.4	(− 0.7 to 4.3)	(− 7.2 to 5.7)	2.3 ± 6.8	3.4	(− 2.8–6.6)	(− 9.1 to 14.2)	0.423
*p*-value*	0.301				0.252				
C7 SVA (mm)									
pre-operative	26.2 ± 22.3	32.9	(16.6–41.9)	(− 17,6 to 57.6)	61.0 ± 32.4	64.6	(43.4–85.5)	(− 2.3 to 109.6)	0.002
Post-operative	0.4 ± 23.7	2.2	(− 18.3to 19.2)	(− 45.5–38.5)	43.0 ± 30.4	42.5	(29.7–62.3)	(− 22.0 to 99.8)	< 0.001
Improvement	− 25.8 ± 28.8	− 15.3	(− 41.7 to − 8.3)	(− 87.9 to 14.9)	− 18.0 ± 30.8	− 24.6	(− 34.3to − 3,9)	(− 71.4 to 44.0)	0.897
*p*-value*	0.001				0.044				
L1-S1 global lordosis (°)									
pre-operative	52.0 ± 9.7	52.8	(42.8–58.2)	(37.9–70.9)	38.4 ± 14.5	40.2	(32.5–46.9)	(1.7–60.9)	0.010
Post-operative	56.5 ± 7.9	57.5	(51.1–62.3)	(39.8–66.7)	43.5 ± 10.9	39.8	(37.9–51.6)	(20.6 –60.2)	< 0.001
Improvement	4.4 ± 6.3	6.0	(− 0.4–8.3)	(− 9.2–15.0)	5.1 ± 8.6	5.3	(− 0.6 to 9.7)	(− 11.8 to 19.0)	0.752
*p*-value*	0.021				0.029				
L4-S1 lordosis (°)									
Pre-operative	18.1 ± 6.8	19.8	(12.7–24.2)	(2.9–26.9)	14.0 ± 7.0	14.0	(10.5–17.4)	(1.8–36.7)	0.014
Post-operative	20.4 ± 7.3	19.3	(16.5–22.3)	(10.7–39.7)	15.3 ± 8.0	14.5	(9.6–17.3)	(16.0–49.0)	0.021
Improvement	2.3 ± 6.9	0.8	(− 2.3 to 7.9)	(− 8.5–15.1)	1.2 ± 7.5	1.9	(− 5.1 to 6.0)	(− 10.6 to 25.6)	0.361
*p*-value*	0.274				0.597				
L5-S1 lordosis (°)									
Pre-operative	18.1 ± 6.8	19.8	(12.7–24.2)	(2.9–26.9)	14.0 ± 7.0	14.0	(10.5–17.4)	(3.4–30.0)	0.119
Post-operative	20.4 ± 7.3	19.3	(16.5–22.3)	(10.7–39.7)	15.3 ± 8.0	14.5	(9.6–17.3)	(3.5–34.6)	0,029
Improvement	2.3 ± 6.9	0.8	(− 2.3 to 7.9)	(− 8.5 to 15.1)	1.2 ± 7.5	1.9	(− 5.1 to 6.0)	(− 9,6 to 16.1)	0.616
*p*-value*	0.274				0.597				
Proximal segmental lordosis** (°)									
Pre-operative	20.2 ± 8.4	17.4	(14.9–26.1)	(8.1–37.9)	16.7 ± 9.9	16.2	(10.4–20.7)	(− 0.1 to 40.7)	0.341
Post-operative	19.0 ± 7.4	18.3	(13.8–22.4)	(8.5–34.8)	13.4 ± 8.3	13.6	(7.0–19.3)	(1.3–30.9)	0.061
Improvement	− 1.2 ± 4.2	− 1.6	(− 3.2 to 0.5)	(− 10.0 to 9.1)	− 3.3 ± 4.8	− 1.9	(− 6.7 to 0.5)	(− 11.7 to 3.6)	0.402
*p*-value*	0,193				0.039				
T1–T12 thoracic kyphosis (°)									
Pre-operative	49.0 ± 12.5	43.6	(38.3–62.3)	(33.6–67.0)	43.8 ± 14.8	38.6	(32.4–54.4)	(18.3–67.1)	0.170
Post-operative	49.8 ± 12.6	46.6	(42.1–59.6)	(27.8–68.0)	45.3 ± 16.1	45.8	(34.9–57.5)	(18.6–70.0)	0.400
Improvement	0.8 ± 3.7	− 0.7	(− 3.8 to 1.4)	(− 6.6 to 5.9)	− 1.5 ± 6.0	− 2.5	(− 4.7 to 1.0)	(− 11.9 to 11.3)	0.539
*p*-value*	0.433				0.193				

*IQR*, interquartile range; ^*^between pre- and post-operative measurements; ^**^at the fusion point

Underlined *p*-values indicate those below 0.05

A greater PT was measured pre-operatively in sitting straight position than in standing position [median: 26.7° (IQR 19, 7–32.2) vs. 15.8° (13.0–25.8), *p* = 0.020] but not post-operatively [24.6° (16.0–30.6) vs. 15.6° (12.4–23.6), *p* = 0.087]. In addition, PT decreased in sitting straight position after lumbar fusion [pre-operative: 26.7° (19.7–32.2) vs. post-operative: 24.6° (16.0–30.6), improvement − 4.3° (− 8.1 to 0.1), *p* = 0.034] but not in standing position [pre-operative: 15.8° (13.0–25,8) vs. post-operative: 15.6° (12.4–23.6), improvement − 0.4° (− 4.4 to 0.8), *p* = 0.245] (Table 1).

When we split subjects with lower instrumented vertebra (LIV) at L5 (10 subjects) from S1 (6 subjects), we did not find any statistically significant difference in PT post-operatively, neither in standing (*p* = 0.368) nor in sitting straight position (*p* = 0.562) (Table 2).

**Table 2:** Post-operative PT and PSL with separation of subjects with LIV L5 versus LIV S1

	Standing posture (*n* = 10 patients)				Sitting posture (*n* = 10 patients)				*p-*value
	Mean ± SD	Median	(IQR)	(Range)	Mean ± SD	Median	(IQR)	(Range)	
Pelvic tilt (°)									
Post-operative LIV LS	18.3 ± 6.5	16.4	(12.9–25.1)	(10.6–27.0)	24.7 ± 110.4	24.6	(16.9–31.3)	(10.6–43.8	0.004
Post-operative LIV SI	15.1 ± 6.6	14.7	(19.9–21.2)	(5.7–23.6)	20.7 ± 11.4	21.8	(9.4–30.4)	(6.3–34.9	0.068
*p*-value*	0.368				0.562				
Proximal segmental lordosis** (°)									
Post-operative LIV L5	18.7 ± 9.3	17.7	(10.6–27.5)	(8.5–34.8)	13.1 ± 8.5	10.6	(7.8–17.3)	(2.4–30.9)	0.002
Post-operative LIV SI	19.5 ± 3.1	20.0	(17.6–22.3)	(14.4–22.8)	13.8 ± 8.7	19.2	(3.9–19.4)	(1.3–19.7)	0.031
*p*-value*	0.492				0.792				

*IQR*, interquartile range; ^*^between LIV at L5 vs. LIV at SI; ^**^at the fusion point Underlined *p*-values indicate those below 0.05; *LIV*, low instrumented vertebra

L1–S1 lordosis (GLL) was greater post-operatively both in standing [pre-operative: 52.8° (42.8–58.2) versus post-operative: 57.5° (51.1–62.3), *p* = 0.021] and sitting straight positions [pre-operative: 40.2° (32.5–46.9) vs. post-operative: 39.8° (37.9–51.6), *p* = 0.029] (Table 1).

**Fig. 2:**
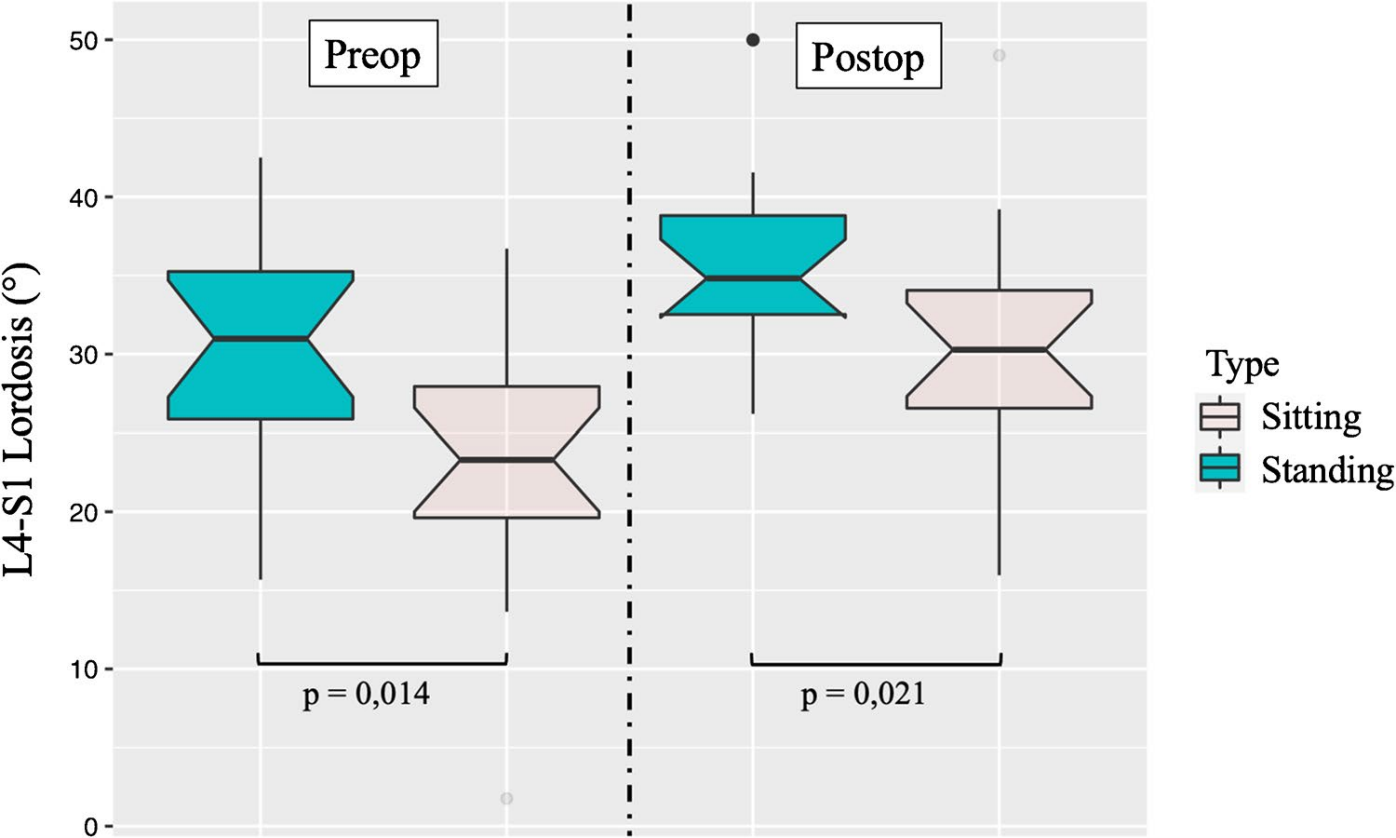
Pre-operative versus post-operative and standing versus sitting straight position for L4-S1 lordosis: significant differences are found between standing and sitting position in pre-operative and post-operative. The plots illustrate median values (boldlines), interquartile ranges (boxes), 95% Cis (whiskers) and outliers (dots)

L4–S1 lordosis was lower in sitting straight than in standing position, both pre-operatively [23.3° (19.6–27.9) vs. 31.0° (25.9–35.3), *p* = 0.014)] and post-operatively [30.3° (26.6–34.1) vs. 34.8° (32.5–38.8), *p* = 0.021]. On the other hand, L4–S1 lordosis increased post-operatively both in standing [31.0° (25.9–35.3) vs. 34.8° (32.5–38.8), *p* = 0.018] and sitting straight position [23.3° (19.6–27.9) vs. 30.3° (26.6–34.1), *p* = 0.004], due to lordosis correction and lumbar instrumentation (Table 1, Fig. 2).

L5–S1 lordosis was only decreased post-operatively between standing and sitting straight position [19.3° (16.5–22.3) vs. 14.5° (9.6–17.3), *p* = 0.029] (Table 1).

**Fig. 3:**
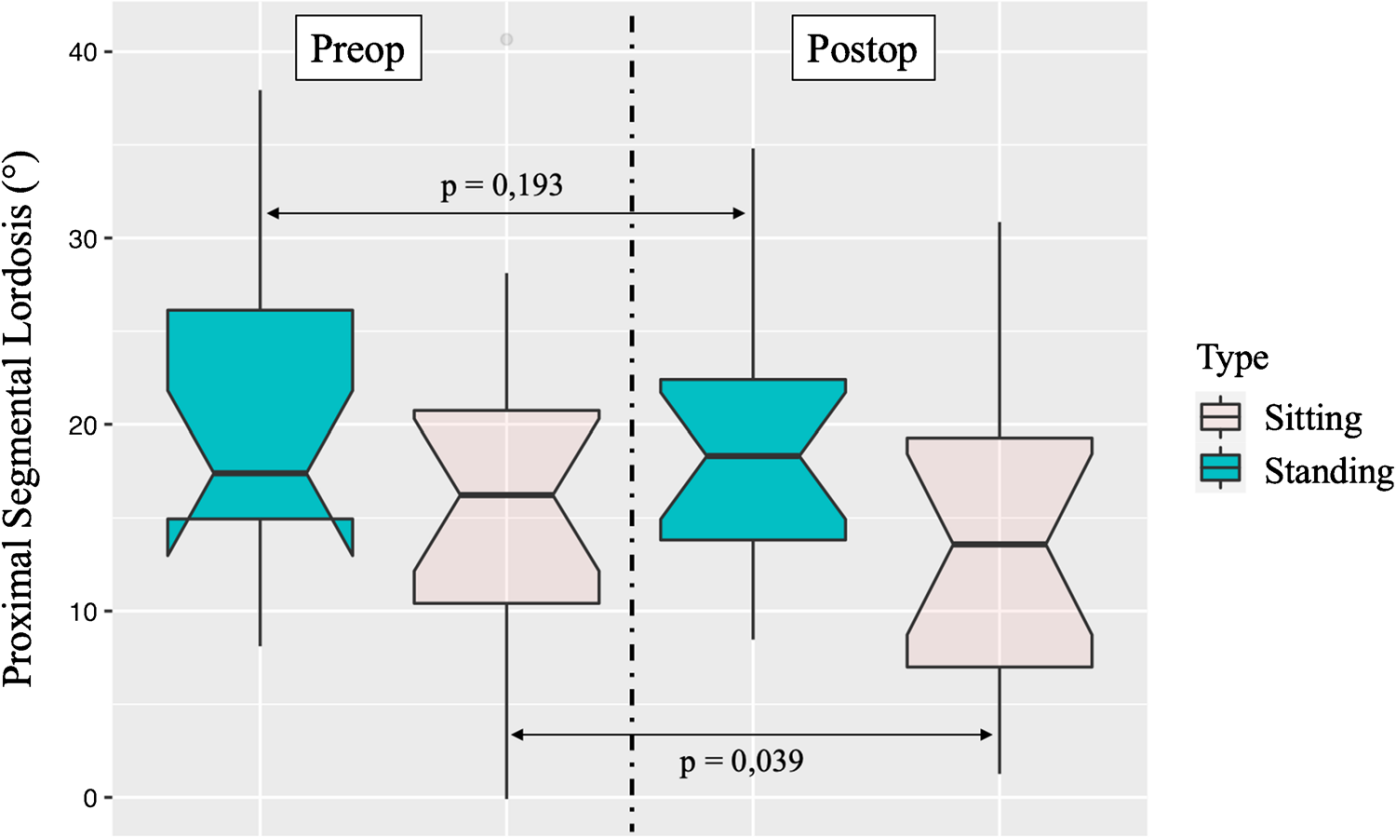
Pre-operative versus post-operative and standing versus sitting straight position for PSL to fusion: a significant difference is found between pre-operative and post-operative for sitting position. The plots illustrate median values (boldlines), interquartile ranges (boxes), 95% Cis (whiskers) and outliers (dots)

Lumbar fusion decreased the PSL in sitting straight position [pre-operative: 16.2° (10.4–20.7) versus post-operative: 13.6° (7.0–19.3), *p* = 0.039] but not in standing position [pre-operative: 17.4 (14.9–26.1) vs. post-operative: 18.3 (13.0.8–22.4), *p* = 0.193] (Table 1, Fig. 3). In most of the patients (75%), the proximal non instrumented segment was L2–L3.

When we compared patients with L5 fusion to those with S1 fusion, we found no difference in PSL post-operatively, neither in sitting straight (*p* = 0.792) nor in standing position (*p* = 0.492) (Table 2).

Only one subject had a neutral PSL morphology (in sitting straight position pre-operatively). In all other subjects, PSL was lordotic (negative value) (Table 3).

**Table 3:** Pre- and post-operative PSL stratified by subjects posture

	Standing posture (*n* = 16)		Sitting posture (*n = 16)	
	*N*	(%)	*N*	(%)
Proximal segmental lordosis (PSL), type of posture				
Pre-operative				
Lordotic	16	(10.0%)	15	(93.8%)
Neutral	0	(0.0%)	1	(6.3%)
Kyphotic	0	(0.0%)	0	(0.0%)
Post-operative				
Lordotic	16	(100.0%)	16	(100.0%)
Neutral	0	(0.0%)	0	(0.0%)
Kyphotic	0	(0.0%)	0	(0.0%)

The C7 SVA was decreased (improved) after fusion both in standing [pre-operative: 32.9 mm (16.6–41.9) vs. post-operative: 2.2 mm (− 18.3 to 19.2), *p* = 0.001] and sitting straight position [pre-operative: 64.6 mm (43.4–85.5) vs. post-operative: 42.5 mm (29.7–62.3), *p* = 0.044]. C7 SVA was smaller in standing than in sitting position both pre-operatively [32.9 mm (16.6–41.9) vs. 64.6 mm (43.4–85.5), *p* = 0,002] and post-operatively [2.2 mm (− 18.3 to 19.2) vs. 42.5 mm (29.7–62.3), *p* < 0.001] (Table 1).

Lumbar apex position was distributed between L2 vertebra and L4–L5 disc pre-operatively for both postures. Post-operatively, the majority of the subjects had an apex located between L4 and L5 vertebrae, both in standing and sitting straight positions.

Finally, no TK differences were observed both in pre-operative versus post-operative and in standing versus sitting straight positions (*p* > 0.05) (Table 2).

## Discussion

Junctional kyphosis is a common post-operative finding in spine surgeries, with incidence ranging from 17 to 61.7% [6–8]. The most frequent type is the proximal junctional kyphosis or PJK, originally described by Glattes et al. in 2005 by a pathologic proximal junctional angle (PJA), defined as a Cobb angle between the inferior endplate of the upper instrumented vertebra (UIV) and the superior endplate of the second vertebra above the UIV, of ≥ 10° and 10° greater than the pre-operative measurement [8]. However, PJK remains a vague entity, as its clinical manifestation can vary from absence of symptoms to invalidating pain, including neurologic signs. Proximal junctional failure (PJF) is considered to be a more severe form of PJK with clinical symptoms and signs, often needing surgical revision [6, 7, 9]. PJF usually appears in the form of a fracture, or a posterior ligamentous disruption, at the UIV or above it. Distal junctional complications have also been described but are less frequent.

Many study groups have tried to elucidate the causes of PJK and PJF, and there is a plethora of articles describing risk factors and suggesting preventive measures during operation [7, 10–13]. It is definitely more interesting to emphasize works related to standing and/or sitting postures, because these junctional mechanical failures have a dynamic origin [14, 15].

For example, Janjua et al. [16] compared standing and sitting radiographs of adult patients operated upon a thoracolumbar deformity. They analysed a group of patients operated upon a thoracolumbar pathology, undergoing a fusion from at least T10 to the lumbar area. They found that a 5° increase in thoracic kyphosis between pre-operative standing and sitting radiographs, predicted a post-operative increase of 6.35° thoracic kyphosis in standing position. This increase in kyphosis is a rebalancing phenomenon.

More recently, Yoshida et al. [17] elaborated a predictive model based on standing versus sitting full spine X-rays. In their retrospective study, they compared pre-operative versus post-operative, standing and sitting slumped positions in ASD patients, and found that the horizontal distance from the UIV to C2 plumb line was a predictive factor for PJK in their series of patients: this distance was larger in patients with PJK than patients without PJK, with a cut-off value at 115 mm. Using this cut-off, they could predict PJK with 90% of sensitivity and 77.1% of specificity. The two groups of patients (with and without PJK) did not show significant differences in the UIV locations.

As shown by previous studies in non-operated subjects, the pelvis retroverts in the sitting straight posture compared to standing, and the lumbar spine adapts by decreasing lordosis mainly at L4–S1 [2–4]. In our small pilot study, we found that thoracolumbar fusion modifies the adaptation of the lumbar spine in sitting position and influences the sagittal alignment. As expected, GLL and L4–S1 increased after fusion in both positions, which was one of the goals of surgical procedure in this series of patients. In addition, the global balance and alignment was improved in standing position after fusion, as demonstrated by the decrease of the C7 SVA value. However, the pelvis cannot retrovert as much as before instrumented fusion when patients change from standing to sitting position (which is an adaptative mechanism in normal spines) as shown by the post-fusion PT decrease in sitting straight, but not in standing position.

In addition, in sitting straight position, the segment immediately above the fusion compensates in flexion (PSL decreases in sitting compared to standing position). This finding suggests that fusing a segment in lordosis, when based on spino-pelvic parameters measured in standing position, results in a kyphosing trend immediately above the UIV when the subject is sitting, which is certainly a cause of locally increased mechanical stress. In this aspect, maintaining a proximal non-fused segment in lordosis (and not in kyphosis) might be protective against increased junctional mechanical stress, but a longer follow-up is needed to demonstrate this. The kyphosing trend above the fusion is also evidenced by a C7 SVA who is greater in sitting than in standing position (both pre- and post-operatively). It suggests that in fused patients, a greater compensation in spinal extension must be performed in sitting position to maintain correct sagittal alignment and preventing the thorax from tilting forward.

Regarding the L5–S1 lordosis, we observe a non-significant decrease (*p* > 0.05) when changing from standing to sitting straight positions in non-fused patients, meaning that the L4–S1 lordosis decrease mainly happens at the L4–L5 level. On the opposite, once fused, we observe a significant L5–S1 lordosis decrease (*p* = 0.029), meaning that due to the rigid instrumentation, the spine compensates in flexion at this level.

When we separate subjects with LIV at L5 versus LIV at S1 post-operatively, we do not observe any significant difference between the two groups for PT and PSL parameters (p > 0.05), but we could hypothesise that patients with fusion including S1 vertebra would have a smaller PT and PSL, due to the lack of compensation in flexion at the L5–S1 level. This parameter will be reanalysed later with a greater number of subjects and a longer follow-up (ongoing prospective study).

Finally, no difference in TK was observed between pre-operative and post-operative, suggesting that the thoracic spine does not influence sagittal compensation and alignment in lumbosacral fusions below L3.

There are several limitations in our study. Due to a small number of study subjects, we cannot at this point make any observation between PI and compensation ability in sitting posture. A longer follow-up is also needed to detect any radiological change in adjacent segments.

From this pilot study, we can conclude that after instrumented fusion, the lumbar spine adapts at extremities of fusion: at the lumbosacral junction and just above the instrumentation (PSL). This mechanism of compensation in anterior flexion seems to be increased in sitting straight posture after a fusion and could favour mechanical complications such as PJK, PJF or even adjacent segment disease (ASD), as these have a similar mechanical origin [15].

We would thus recommend to pay attention to several aspects when considering lumbar fusion:

- Lordosis should be carefully corrected/restored at the L4–S1 levels.
- Avoid correcting lordosis above the L4 level, as this will lead to proximal lumbar hyperlordosis, which then needs to be compensated in anterior flexion, especially in sitting position. [14, 18]
- When possible, do not fuse the L5–S1 level, as it is an important hinge level for the sitting posture (we would recommend not to fuse up to modified Pfirrmann grade 4 at least).
- Use planning softwares (many available) to simulate the lordosis correction using standing and sitting full spine X-rays, considering that the ideal lumbar lordosis would probably be intermediate between standing and sitting values.

The goal of all these measures, would be to maintain the segment immediately above the fusion in lordosis when sitting, as this certainly protects from junctional mechanical overstress. Further studies are needed to understand precisely what factors are involved in PJK/PJF, which is an acute mechanical complication, versus ASD, which is slower and can manifest as a progressive disc degeneration or vertebral wedging.

This field of research promises to be very exciting, and we are convinced that it will be the subject of many other publications in the near future.

## Data Availability

The datasets generated during and/or analysed during the current study are available from the corresponding author on reasonable request.

## References

[1] Baumgartner D, Zemp R, List R, Stoop M, Naxera J, Elsig JP, et al., (2012) The spinal curvature of three different sitting positions analysed in an open MRI scanner. ScientificWorldJournal, 2012 18401610.1100/2012/184016 10.1100/2012/184016 PMC3512264 23226980

[2] Hey HW, Wong CG, Lau ET, Tan KA, Lau LL, Liu KG (2017). Differences in erect sitting and natural sitting spinal alignment-insights into a new paradigm and implications in deformity correction. Spine J.

[3] Hey HWD, Lau ET, Tan KA, Lim JL, Choong D, Lau LL (2017). Lumbar spine alignment in six common postures: an ROM analysis with implications for deformity correction. Spine (Phila Pa 1976).

[4] Hey HWD, Teo AQA, Tan KA, Ng LWN, Lau LL, Liu KG (2017). How the spine differs in standing and in sitting-important considerations for correction of spinal deformity. Spine J.

[5] Le Huec JC, Thompson W, Mohsinaly Y, Barrey C, Faundez A (2019). Sagittal balance of the spine. Eur Spine J.

[6] Lee J, Park YS (2016). Proximal junctional kyphosis: diagnosis, pathogenesis, and treatment. Asian Spine J.

[7] Kim HJ, Iyer S (2016). Proximal Junctional Kyphosis. J Am Acad Orthop Surg.

[8] Glattes RC, Bridwell KH, Lenke LG, Kim YJ, Rinella A, Edwards C (2005). Proximal junctional kyphosis in adult spinal deformity following long instrumented posterior spinal fusion: incidence, outcomes, and risk factor analysis. Spine (Phila Pa 1976).

[9] Yagi M, Rahm M, Gaines R, Maziad A, Ross T, Kim HJ (2014). Characterization and surgical outcomes of proximal junctional failure in surgically treated patients with adult spinal deformity. Spine (Phila Pa 1976).

[10] Line BG, Bess S, Lafage R, Lafage V, Schwab F, Ames C (2020). Effective prevention of proximal junctional failure in adult spinal deformity surgery requires a combination of surgical implant prophylaxis and avoidance of sagittal alignment overcorrection. Spine (Phila Pa 1976).

[11] Daniels AH, Reid DBC, Durand WM, Line B, Passias P, Kim HJ, et al., (2020) Assessment of patient outcomes and proximal junctional failure rate of patients with adult spinal deformity undergoing caudal extension of previous spinal fusion. World Neurosurg e449-e454. 10.1016/j.wneu.2020.04.024 10.1016/j.wneu.2020.04.024 32305603

[12] Safaee MM, Deviren V, Dalle Ore C, Scheer JK, Lau D, Osorio JA (2018). Ligament augmentation for prevention of proximal junctional kyphosis and proximal junctional failure in adult spinal deformity. J Neurosurg Spine.

[13] Ghobrial GM, Eichberg DG, Kolcun JPG, Madhavan K, Lebwohl NH, Green BA (2017). Prophylactic vertebral cement augmentation at the uppermost instrumented vertebra and rostral adjacent vertebra for the prevention of proximal junctional kyphosis and failure following long-segment fusion for adult spinal deformity. Spine J.

[14] Faundez AA, Richards J, Maxy P, Price R, Leglise A, Le Huec JC (2018). The mechanism in junctional failure of thoraco-lumbar fusions. Part II: Analysis of a series of PJK after thoraco-lumbar fusion to determine parameters allowing to predict the risk of junctional breakdown. Eur Spine J.

[15] Le Huec JC, Richards J, Tsoupras A, Price R, Leglise A, Faundez AA (2018). The mechanism in junctional failure of thoraco-lumbar fusions. Part I: Biomechanical analysis of mechanisms responsible of vertebral overstress and description of the cervical inclination angle (CIA). Eur Spine J.

[16] Janjua MB, Tishelman JC, Vasquez-Montes D, Vaynrub M, Errico TJ, Buckland AJ (2018). The value of sitting radiographs: analysis of spine flexibility and its utility in preoperative planning for adult spinal deformity surgery. J Neurosurg Spine.

[17] Yoshida G, Ushirozako H, Hasegawa T, Yamato Y, Kobayashi S, Yasuda T, et al., (2020) Preoperative and postoperative sitting radiographs for adult spinal deformity surgery: upper instrumented vertebra selection using sitting C2 plumb line distance to prevent proximal junctional kyphosis. Spine (Phila Pa 1976 E950-E958. 10.1097/brs.0000000000003452 10.1097/BRS.0000000000003452 32675610

[18] Yilgor C, Sogunmez N, Boissiere L, Yavuz Y, Obeid I, Kleinstück F (2017). Global alignment and proportion (GAP) score: development and validation of a new method of analyzing spinopelvic alignment to predict mechanical complications after adult spinal deformity surgery. J Bone Joint Surg Am.

